# All Eyes on the New, but Who Hears the Old? The Impact of Incumbent Employees’ Perceived Status Threat on Work Behavior

**DOI:** 10.3390/bs15111550

**Published:** 2025-11-13

**Authors:** Yanshu Ji, Ke Hu, Wen Zhang, Yuanyun Yan

**Affiliations:** 1 School of Business, Nanjing University, Nanjing 210093, China; yanshuji@smail.nju.edu.cn (Y.J.);; 2 School of Entrepreneurship, Zhejiang University of Finance and Economics Dongfang College, Haining 314408, China; 3 School of Finance, Southwestern University of Finance and Economics, Chengdu 610074, China; 4 School of Economics and Management, Neijiang Normal University, Neijiang 641112, China

**Keywords:** perceived status threat, cognitive appraisal theory of stress, coping strategy, newcomer socialization

## Abstract

This research applies the stress appraisal framework to examine how perceived status threats, triggered by high-performing new employees, affect incumbent employees’ work engagement and withdrawal behaviors. The investigation proposes that coping approaches, specifically proactive adaptation strategies and disengagement tactics, serve as mediating mechanisms, with stress perception orientation playing a moderating role. By reversing traditional research perspectives to concentrate on incumbent employees rather than new employees, this analysis identifies the key drivers of perceived occupational vulnerability and investigates their behavioral consequences. Through a time-lagged research methodology, we garnered responses from 266 incumbent employees spanning multiple sectors. The results demonstrate a strong positive correlation between the competence of new employees and incumbent employees’ perceived status of threat, which subsequently elevates work engagement via approach-focused strategies, while simultaneously increasing disengagement behaviors through avoidance mechanisms. Notably, employees’ fundamental beliefs about stress significantly weaken the association between perceived competitive threats and passive coping methods. These discoveries highlight critical implications for managing workplace dynamics and optimizing team performance through an enhanced understanding of perceived status challenges.

## 1. Introduction

The pursuit of social standing represents a fundamental human motivation (Anderson et al., 2012; Bendersky & Pai, 2018), with one’s hierarchical position critically shaping behavioral patterns (Yu & Duffy, 2016; Zhou et al., 2022). Attaining higher status grants individuals amplified visibility along with privileged access to strategic information and prospects (Berger et al., 1980; Foschi, 2000; Ridgeway & Correll, 2006). Nevertheless, status represents a finite positional resource characterized by zero-sum dynamics, wherein advancement for some necessitates relative decline for others (Bendersky & Hays, 2012). This competitive reality compels active status-seeking behaviors, while simultaneously exposing individuals to a potential decline in status compared to competitors (Khan et al., 2018; Kellogg, 2012). Conceptualizations of perceived status threat involve organizational members’ recognition that their social standing is being contested, potentially diminishing their authority, prestige, and esteem (Aquino & Douglas, 2003; Ridgeway, 1982). The current organizational behavior literature predominantly examines the consequences of such perceptions, particularly employees’ reactive strategies to protect their status (Bai, 2017; Pettit et al., 2010; Yu et al., 2018). While some researchers emphasize beneficial outcomes such as enhanced ethical conduct (G. Zhang et al., 2020) and prosocial behavior (Hays & Blader, 2017), others document detrimental effects, including supervisory abuse (Khan et al., 2018), interpersonal sabotage (Reh et al., 2018), and colleague undermining (Sun, 2022).

Notwithstanding these advancements, several critical gaps persist in status threat research. Primarily, existing investigations inadequately address the origins of status threats within organizational hierarchies. The current scholarship disproportionately examines leader–subordinate dynamics (Khan et al., 2018; G. Zhang et al., 2020) or peer-level rivalries (Campbell et al., 2017; Reh et al., 2018), neglecting the potential impact of high-performing new employees on incumbent employees’ status perceptions. Secondarily, the extant studies predominantly examine either beneficial or harmful effects in isolation (Sun, 2022; N. Zhang et al., 2023), producing inconsistent findings without comprehensive frameworks explaining dual-effect manifestations. Finally, while employee psychological diversity represents a key organizational reality (N. Zhang et al., 2023), the current research insufficiently explores how individual differences moderate responses to status threats.

This study is based on the stress cognitive appraisal framework (Lazarus & Folkman, 1984, 1987) to analyze incumbents’ behavioral reactions to status threats. Central to this theory is how individuals evaluate stressors. In the primary appraisal phase, they assess whether environmental demands affect their well-being, which can be appraised as neutral, beneficial, or threatening (Lazarus & Folkman, 1984). Threat-based appraisals focus on the negative consequences of stressors, prompting defensive coping strategies (e.g., avoidance) to alleviate psychological discomfort. In the secondary appraisal phase, individuals assess whether they have sufficient resources to cope with the threat, which further influences their coping strategy choices (Jiang et al., 2021; Bliese et al., 2017). Based on this framework, we hypothesize that new employees’ superior task performance acts as a stressor that triggers incumbents’ perceived status threat, which subsequently affects their work-related behaviors through primary threat-based appraisal and secondary coping strategy selection.

The sample consisted of 266 incumbent employees from various industries who worked alongside newly hired colleagues. It was demographically diverse in terms of age, tenure, job level, and organizational type. Using a two-wave three-week lagged survey design, this study reduced potential common-method bias and ensured sample diversity across organizations. Our results showed that new employees’ exceptional task performance was positively associated with incumbents’ perceived status threat. This perception, in turn, exerted a dual impact on work behaviors: it promoted work engagement through approach coping strategies (e.g., task involvement) and encouraged work withdrawal through avoidance coping strategies (e.g., disengagement). Furthermore, a stress mindset moderated these relationships, with a growth-oriented mindset attenuating the direct link between status threat and avoidance responses and weakening the indirect path from perceived threat to work withdrawal through avoidance coping.

This research offers three primary theoretical contributions. First, it identifies new employees’ exceptional task performance as a key antecedent of incumbents’ perceived status threat, thereby extending the literature on status threat from interpersonal perceptions to team-level adaptation dynamics. By linking status threat to both work engagement and withdrawal, this study clarifies its dual motivational consequences and deepens theoretical insight into how status concerns shape employee behaviors within teams. Second, it reframes research on workplace new employee dynamics by shifting the focus from new employees’ adjustment to incumbents’ adaptive responses. This perspective highlights the interactive nature of status threat, emphasizing that the integration of new employees is not a one-sided adjustment process but a reciprocal adaptation that also triggers status-related reactions among incumbent employees. Third, the study extends the stress appraisal framework by elucidating how incumbents evaluate and cope with status-related stressors. It further identifies a stress mindset as a critical boundary condition that influences how status threat is appraised and translated into behavior. This integration advances our theoretical understanding of the cognitive and motivational mechanisms underlying employees’ responses to status-challenging situations.

## 2. Theory and Hypotheses

### 2.1. Cognitive Appraisal Theory of Stress

This research is founded on Lazarus and Folkman’s (1987) stress cognitive evaluation framework, which emphasizes distinct individual responses to stressors based on personal characteristics and situational factors, ultimately shaping diverse adaptive behaviors. At the primary evaluation phase, people determine whether environmental demands affect their welfare, with possible assessments being neutral, beneficial, or stress-inducing (differentiated into development-focused and threat-based evaluations). Development-focused evaluation involves perceiving stressors through the lens of possible achievement and self-improvement, motivating proactive engagement strategies that enhance personal resources or modify environmental challenges. By contrast, threat-based evaluation centers on perceived dangers and negative consequences, prompting defensive mechanisms aimed at mitigating psychological discomfort (Lazarus & Folkman, 1984; Jiang et al., 2021; Bliese et al., 2017). This conceptual model offers an operational paradigm for analyzing behavioral responses under conditions of pressure.

**Figure 1:**
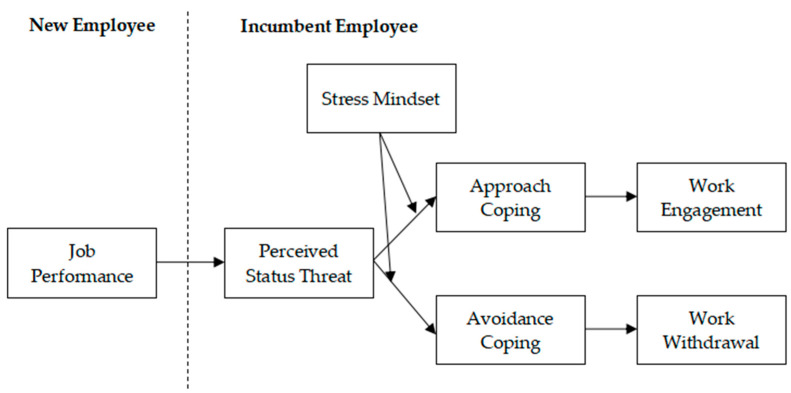
Theoretical model.

Building upon the cognitive appraisal theory of stress (Lazarus & Folkman, 1984, 1987), the present study applied the two-stage appraisal logic to explain how incumbents cognitively interpret and behaviorally respond to new employees’ exceptional performance. During primary appraisal, incumbents evaluate the new employees’ performance as either a developmental opportunity (challenge appraisal) or a potential threat to their status (threat appraisal). Subsequently, in secondary appraisal, they assess their perceived control and coping resources—such as professional competence, tenure-based expertise, or social support—to determine feasible coping actions. These two appraisal stages jointly shape whether incumbents adopt approach-oriented strategies to restore or improve their standing or avoidance-oriented strategies to minimize threat exposure. Thus, cognitive appraisal theory provides the overarching mechanism linking perceived status threat, coping strategies, and behavioral outcomes in this research model. Accordingly, our model explicitly maps primary threat appraisal (triggered by new employees’ high performance) to perceived status threat and secondary appraisal to the selection of approach versus avoidance coping, which in turn leads to divergent work outcomes (see Figure 1 for our research model).

### 2.2. New Employees’ Job Performance and Incumbent Employees’ Perceived Status Threat

In workplace environments, employees’ sense of status vulnerability often stems from comparative job achievements among colleagues (Aquino & Douglas, 2003; Davis, 2015; Reh et al., 2018). The research indicates that professional accomplishments significantly influence one’s standing within organizational hierarchies (Lee et al., 2018), with higher levels of work output typically correlating with elevated organizational rank (Brown et al., 2007). Within competitive workplace dynamics, incumbent employees continually assess both their personal progress and that of their peers (Reh et al., 2018). When observing superior performance from new employees, this comparison may trigger concerns about potential replacement scenarios, leading to heightened stress regarding the preservation of one’s current hierarchical position. Perceived status threat is defined as an employee’s belief that their relative standing, prestige, or influence within a group is being challenged by others (Aquino & Douglas, 2003; Bendersky & Hays, 2012). It reflects a psychological state of status vulnerability arising from social comparisons or competitive performance contexts. From a cognitive appraisal perspective, such comparative evaluations represent primary appraisals in which incumbents interpret new employees’ outstanding performance as a possible status threat rather than a benign event. This threat appraisal activates stress-related cognitive and emotional reactions that form the psychological basis of perceived status threat. Based on these observations, we propose the following hypothesis:

**H1.**  *New employees’ job performance is positively correlated with incumbent employees’ perceived status threat.*

### 2.3. Incumbent Employees’ Perceived Status Threat and Work Behavior

Within organizational environments, workers initiate behavioral responses to protect their hierarchical standing when they sense their hierarchical standing is at risk (Bai, 2017; Pettit et al., 2010; Yu et al., 2018). Proactive measures might be adopted by staff to preserve or elevate their workplace position. Research by Hays and Blader (2017) revealed that elite organizational members experiencing doubts about their status legitimacy tend to demonstrate heightened benevolence toward junior colleagues to reestablish equitable conditions. Additionally, counterproductive strategies may emerge through attempts to diminish peers’ professional standing. Khan et al. (2018) observed that supervisors prioritizing hierarchical maintenance might interpret exceptional subordinate performance as status ambiguity triggers, potentially manifesting in hostile managerial conduct. Current academic discourse tends to examine either beneficial or detrimental outcomes separately, with limited exploration of their simultaneous occurrence.

Perceived status threat constitutes a salient social-evaluative stressor (Bendersky & Hays, 2012; Reh et al., 2018). According to cognitive appraisal theory, such stressors evoke a sequential evaluative process: in the primary appraisal, incumbents perceive potential damage to self-worth and social position, while in the secondary appraisal, they assess coping efficacy and select behavioral responses accordingly. When resources are deemed sufficient in the secondary appraisal, employees adopt approach coping—problem-focused strategies such as knowledge sharing or skill enhancement (Carver & Connor-Smith, 2010)—which promote work engagement (i.e., the vigorous investment of physical, cognitive, and emotional energies in one’s tasks; Kahn, 1990) through motivational resource gain (Bakker & Demerouti, 2017; Cavanaugh et al., 2000). Conversely, when the secondary appraisal indicates resource insufficiency, employees tend to adopt avoidance coping—withdrawal or detachment to reduce stress exposure (Halbesleben et al., 2014)—thereby fostering work withdrawal (e.g., psychological disengagement, lateness, or absenteeism; Lehman & Simpson, 1992; Nauman et al., 2020).

Hence, integrating cognitive appraisal logic, we propose that incumbent employees’ perception of status risks from high-achieving new employees generates dual behavioral consequences. Primarily, such perceived challenges may enhance occupational commitment, characterized by vigorous investment of physical, cognitive, and affective resources in professional tasks (Kahn, 1990). Motivated by status preservation, incumbent employees might demonstrate enhanced initiative through visible organizational contributions and expertise display (Berger et al., 1972; Anderson & Kilduff, 2009; Ridgeway & Berger, 1986; Bai, 2017). Contrastingly, the study concurrently suggests these perceived threats may amplify disengagement patterns, encompassing psychological detachment or physical withdrawal behaviors such as task postponement and attendance irregularities (Lehman & Simpson, 1992; Nauman et al., 2020). Tenured personnel accustomed to hierarchical deference (Blader & Chen, 2012; Farh et al., 2007; Marr & Thau, 2014) might interpret perceived status erosion as professional disrespect, eliciting emotional distress that manifests in reduced work participation and occupational exhaustion (Anderson et al., 2015; Harvey et al., 2007). Our theoretical framework proposes the following hypotheses:

**H2a.**  *Incumbent employees’ perceived status threat is positively correlated with work engagement.*

**H2b.**  *Incumbent employees’ perceived status threat is positively correlated with work withdrawal behavior.*

### 2.4. The Mediating Role of Approach Coping and Avoidance Coping

The anxiety stemming from perceived status threats caused by new employees’ superior performance serves as a critical workplace stressor for existing staff. Within the framework of cognitive appraisal theory, coping represents the behavioral manifestation of the secondary appraisal—reflecting how individuals translate evaluative outcomes into action. Specifically, approach coping is the behavioral instantiation of challenge-consistent secondary appraisal, whereas avoidance coping reflects threat-consistent secondary appraisal. When workers perceive sufficient capability and resources to manage the threat while anticipating professional development through status competition, they frame it as a developmental challenge (Rodell & Judge, 2009). Approach and avoidance coping represent two broad response orientations toward stressors (Carver & Connor-Smith, 2010; Bliese et al., 2017). Approach coping entails problem-focused and engagement strategies that seek to resolve the stressor or improve one’s resources, whereas avoidance coping involves emotion-focused and withdrawal strategies that minimize exposure to the stressor without addressing its source. Consistent with the approach–avoidance model (Carver & White, 1994; Lazarus & Folkman, 1984), employees may react to perceived status threat through either energized engagement or defensive withdrawal.

A positive appraisal fosters future-oriented optimism, prompting proactive strategies such as utilizing organizational knowledge (Burris, 2012), initiating problem-solving approaches, and enhancing task commitment to demonstrate one’s organizational value, thereby mitigating stress consequences. Contrarily, when perceiving inadequate competencies, employees dread potential status erosion and resource depletion in competitive scenarios, framing the situation as career obstruction (Khan et al., 2018). A negative appraisal generates replacement anxiety and status-loss fears, triggering defensive responses such as selective information filtering and stressor disengagement (Anshel et al., 2010). This manifests as cognitive detachment through workplace inattention and behavioral disengagement, including absenteeism or turnover intentions. We therefore propose the following hypotheses:

**H3a.**  *Approach coping mediates the relationship between incumbent employees’ perceived status threat and work engagement.*

**H3b.**  *Avoidance coping mediates the relationship between incumbent employees’ perceived status threat and work withdrawal.*

### 2.5. The Moderating Role of Stress Mindset

Stress mindset refers to the mental framework individuals hold about the potential of stress to improve or impair performance, health outcomes, and personal development, remaining unaffected by personal requirements or available assets (Crum et al., 2017). In other words, it reflects individuals’ generalized beliefs about whether stress is enhancing or debilitating (Crum et al., 2013, 2017). Those with an enhancing orientation view stress as a catalyst for growth and learning, whereas those with a debilitating orientation perceive stress as harmful and to be avoided.

The stress mindset has two qualitatively distinct orientations—growth-oriented versus limiting perspectives—that shape how individuals appraise and respond to stressors (Crum et al., 2017). Accordingly, when employees hold a growth-oriented mindset, they are more likely to interpret stress as an opportunity for mastery and improvement; when they hold a limiting mindset, they are more likely to see stress as a threat that constrains performance. This “when–such that” conceptualization provides a boundary condition lens through which the coping responses to status threat can be understood (Karampas et al., 2020).

Integrating a stress mindset into the cognitive appraisal framework provides a theoretical extension by emphasizing how enduring belief systems shape primary and secondary appraisals. Specifically, employees with a growth-oriented stress mindset are more likely to interpret status threats as development-focused challenges during the primary appraisal and perceive greater control and coping resources during the secondary appraisal, leading to adaptive and approach-oriented coping. Conversely, those with a limiting stress mindset tend to appraise the same stressor as a severe threat, experience restricted coping resources, and thus engage in avoidance coping.

Building on this reasoning, we expect that when employees adopt a more growth-oriented stress mindset, the positive relationship between perceived status threat and approach coping will be stronger, whereas when they hold a more limiting stress mindset, the positive relationship between perceived status threat and avoidance coping will be stronger. Building on these theoretical foundations, we propose the following hypotheses:

**H4a.**  *The stress mindset positively moderates the relationship between incumbent employees’ perceived status threat and approach coping. The stronger the stress mindset, the stronger the relationship.*

**H4b.**  *The stress mindset negatively moderates the relationship between incumbent employees’ perceived status threat and avoidance coping. The stronger the stress mindset, the weaker the relationship.*

**H5a.**  *The stress mindset positively moderates the indirect effect of incumbent employees’ perceived status threat on work engagement through approach coping. The stronger the stress mindset, the stronger the indirect effect.*

**H5b.**  *The stress mindset negatively moderates the indirect effect of incumbent employees’ perceived status threat on work withdrawal through avoidance coping. The stronger the stress mindset, the weaker the indirect effect.*

## 3. Methods

### 3.1. Sample and Procedure

Data were collected via Sojump (www.sojump.com) (Del Ponte et al., 2024), a professional online survey platform frequently used in scholarly research. All participants provided informed consent, and duplicate IPs were screened to ensure data quality. The respondents were instructed to identify one recently hired colleague with whom they had collaborated closely for at least one month. To minimize bias, we controlled for demographic and positional variables and employed a two-wave (three-week-lagged) design. This procedure reduces potential third-variable and common-method concerns while maintaining sample diversity across organizations.

The research further employed an extended timeframe to categorize new versus established workforce members following Gong et al.’s (2009) framework. Participants were divided into two groups based on tenure length: those with under three years’ employment were classified as new employees, while incumbents were defined as having completed at least three years of service. To address potential common method variance, a dual-phase survey design was implemented with a 14-day interval between administrations. The initial data collection yielded 572 usable questionnaires, capturing new employees’ job performance metrics, incumbents’ perceived status challenges, adaptive strategies (both proactive and evasive), and stress perception patterns. Subsequent follow-up surveys specifically examined incumbent employees’ work involvement and disengagement behaviors, exclusively targeting first-phase respondents. The final dataset comprised 266 qualified responses.

Among the samples, females accounted for 52.26% of the respondents. Approximately 51.88% of employees were aged 26–35 years. A total of 79.32% held a bachelor’s degree or above. Employees with 3–6 years of work experience constituted the largest group, representing 63.53% of the sample. Regarding job level, 42.48% were general staff, while 57.52% held junior, middle, or senior management positions. As for organizational type, private enterprises comprised the majority, accounting for 56.39% of the respondents. A detailed frequency analysis of demographic variables is presented in Table 1.

### 3.2. Measurements

The measurement tools employed in this investigation were adapted from existing scholarly sources. To mitigate potential cultural interpretation discrepancies, comprehensive translation–backtranslation procedures were implemented for all survey components. Responses were collected using a five-point Likert-type metric anchored at opposing extremes (1 = strongly disagree to 5 = strongly agree).

New Employees’ Job Performance: New employees’ work outcomes were evaluated using Gong et al.’s (2009) four-item metric. Representative question: “The recent hire consistently exceeds expected productivity benchmarks” (α = 0.86).

Incumbent Employees’ Perceived Status Threat: Incumbent employees’ status concerns were operationalized through a modified three-item instrument adapted from Okimoto and Wenzel’s (2011) original scale. Illustrative item: “How significantly does the new colleague’s achievement affect your standing within the organizational hierarchy?” (α = 0.79).

Approach Coping and Avoidance Coping: Drawing upon Anshel et al.’s (2010) framework, six indicators assessed adaptive and evasive coping strategies. Active response example: “I strategize practical solutions to address status-related challenges” (α = 0.91). Avoidance behavior illustration: “I engage in diversionary activities to minimize stress exposure” (α = 0.77).

Stress Mindset: Psychological responses to pressure situations were quantified using Crum et al.’s (2013) eight-dimensional assessment. Characteristic statement: “Stressful experiences facilitate personal development and skill acquisition” (α = 0.88).

Work Engagement: The investigation utilized the three-dimensional framework (vigor, dedication, and absorption) refined by Schaufeli et al. (2006), comprising nine total indicators. Sample descriptor: “My professional role consistently energizes me” (α = 0.91).

Work Withdrawal: Workplace disengagement patterns were measured through Lehman and Simpson’s (1992) twelve-item scale, evaluating attitudinal disengagement and observable withdrawal behaviors. Example behavior: “Occasionally underperforming task requirements” (α = 0.88).

Control Variables: Following established methodological protocols (Lang et al., 1990), participant characteristics, including biological sex, chronological age, and educational attainment, were incorporated. The empirical evidence suggests that the organizational tenure (Zhou et al., 2020) and hierarchical position influence resource allocation dynamics, while institutional type modulates competitive environments and incentive structures (Li et al., 2021). Accordingly, these variables were included in the analytical models.

## 4. Results

### 4.1. Confirmatory Factor Analysis

Prior to hypothesis testing, the measurement model was re-estimated to confirm the construct validity and alignment with the revised conceptual framework. Following the theoretical refinements and construct re-specifications, we re-estimated the measurement model to ensure consistency with the updated conceptual framework. Confirmatory factor analysis (CFA) was conducted using AMOS 24.0 to evaluate the construct validity of seven core variables. To minimize intergroup disparities, an equilibrium approach within the item-parceling framework was employed, grouping items by factor loadings (Hayes & Preacher, 2013). The seven-factor measurement model included newcomer task proficiency, incumbents’ perceived occupational jeopardy, active adaptation strategies, passive response mechanisms, stress mindset, job engagement, and work withdrawal.

As shown in Table 2, the model adequacy was assessed using multiple fit indices, including χ^2^, the Tucker–Lewis index (TLI), the comparative fit index (CFI), the root mean square error of approximation (RMSEA), and the standardized root mean square residual (SRMR). The results indicated a satisfactory model fit (χ^2^ = 486.93, df = 301, CFI = 0.95, TLI = 0.95, RMSEA = 0.05, SRMR = 0.06). All standardized factor loadings were above 0.65 (*p* < 0.001). The composite reliability values ranged from 0.77 to 0.90, and the average variance extracted (AVE) values ranged from 0.61 to 0.79, surpassing the recommended thresholds (Fornell & Larcker, 1981), confirming good convergent validity for the key variables in the model.

To further examine discriminant validity, this study followed the method proposed by Fornell and Larcker (1981) and analyzed the average variance extracted (AVE) for the seven key variables, as shown in Table 3. The square roots of the AVE for all seven key variables were higher than the correlations between these variables, providing further evidence of good discriminant validity among the core constructs in this study.

### 4.2. Common Method Variance Tests

While this investigation gathered responses across two temporal intervals, potential common method bias remained a consideration due to the exclusive reliance on employee self-reports. To assess the measurement integrity and data trustworthiness, an initial application of Harman’s single-factor analysis was implemented. The findings indicated that the primary unrotated factor accounted for merely 23.33% of the total variance, remaining substantially below the 40% cutoff established by Hayes and Preacher (2013). Further supporting this conclusion, the confirmatory factor analysis outcomes presented in Table 1 revealed strong discriminant validity across all seven measured variables. These multiple validation approaches collectively confirmed that significant common method bias did not substantially affect this investigation.

### 4.3. Descriptive Analyses

The statistical analysis outcomes presented in Table 3 reveal important variable relationships. The initial examination demonstrated a statistically significant positive connection between new employees’ work effectiveness and incumbent employees’ perception of status risks (r = 0.238, *p* < 0.001). Subsequent findings indicated that incumbent employees’ status concerns showed strong positive correlations with both proactive strategies (r = 0.481, *p* < 0.001) and evasive behaviors (r = 0.145, *p* = 0.018). Further analysis confirmed that active coping mechanisms substantially predicted increased work commitment (r = 0.425, *p* < 0.001), while avoidance responses were significantly associated with occupational disengagement tendencies (r = 0.350, *p* < 0.001). These patterns offer empirical validation for the conceptual framework guiding this investigation, establishing a foundation for subsequent hypothesis verification processes.

### 4.4. Hypothesis Testing

#### 4.4.1. Main Effects and Mediating Effects

Table 4 displays the results of the hierarchical regression analysis. New employees’ work effectiveness exhibited a pronounced positive association with incumbent employees’ perception of status risks (Model 2, β = 0.26, *p* < 0.001), thereby validating Hypothesis 1. The perceived status insecurity among incumbent employees demonstrated a significant relationship with enhanced occupational commitment (Model 4, β = 0.278, *p* < 0.001), confirming Hypothesis 2a. Concurrently, status-related apprehensions substantially correlated with increased workplace disengagement behaviors (Model 7, β = 0.11, *p* < 0.001), aligning with Hypothesis 2b.

Upon integrating active adaptation and passive response strategies as mediating factors, the analysis revealed that proactive coping mechanisms positively influenced work dedication (Model 5, β = 0.34, *p* < 0.001). Subsequently, the previously observed connection between status perception and engagement diminished (Model 5, β = 0.10, *p* = 0.098), suggesting complete mediation through approach-oriented strategies and substantiating Hypothesis 3a. Bootstrap analysis (PROCESS Model 4) quantified this indirect effect at 0.180, with 95% CI [0.111, 0.256] excluding null values as corroborated by Hayes (2012).

Parallel findings emerged regarding withdrawal tendencies, where evasive coping patterns significantly predicted disengagement behaviors (Model 8, β = 0.24, *p* < 0.001). The original impact of status perception diminished (Model 8, β = 0.08, *p* = 0.003), indicative of complete mediation and supporting Hypothesis 3b. Subsequent bootstrap testing measured the indirect pathway through avoidance strategies at 0.031 (95% CI [0.002, 0.068]), with confidence boundaries excluding zero as per methodological guidelines (Hayes, 2012).

#### 4.4.2. Moderating Effects and Moderated Mediation Effects of the Stress Mindset

**Figure 2:**
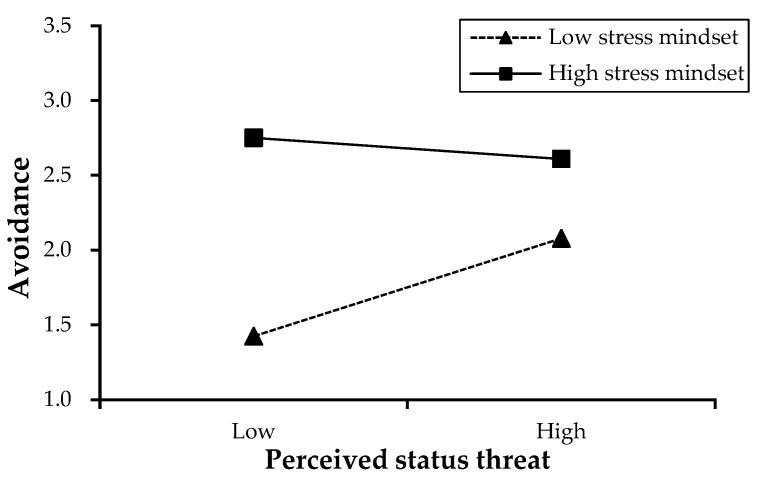
The moderating effect of stress mindset.

To examine the moderating role of the stress mindset, we developed a multiplicative interaction term combining employees’ perceived status threat measurements with their stress mindset scores for stepwise hierarchical regression modeling. The analytical outcomes presented in Table 4 demonstrate nonsignificant moderation effects on approach-oriented strategies (Model 11: β = −0.07, *p* = 0.363), thereby rejecting Hypotheses 4a and 5a. Conversely, the interaction exhibited a statistically meaningful negative relationship with avoidance coping mechanisms (Model 14: β = −0.20, *p* = 0.003), confirming Hypothesis 4b. Through visual representation using ±1 SD adjustments based on Aiken and West’s (1991) analytical framework (Figure 2), distinct patterns emerged. The graphical analysis revealed a marked positive association between perceived status threat and avoidance behaviors under low stress mindset conditions (β = 0.33, *p* = 0.003), whereas no meaningful connection was observed when stress mindset levels were elevated (β = −0.07, *p* = 0.210). This differential pattern reinforces the hypothesized moderating effect on avoidance strategies, highlighting the conditional nature of status threat impacts depending on cognitive appraisal patterns.

To comprehensively examine moderated mediation dynamics, the research implemented Hayes’s bootstrap resampling technique (PROCESS Model 7) to assess coping avoidance’s mediation across varying stress mindset intensities. The analysis revealed that under minimal stress mindset conditions, the perceived status threat’s mediation pathway through avoidance coping yielded a coefficient of 0.062 (95% CI [0.022, 0.117]), with interval boundaries excluding null values. Conversely, elevated stress mindset levels rendered this mediation insignificant (95% CI [−0.032, 0.029]), maintaining the exclusion of zero. The contrast between these pathways reached −0.047 (95% CI [−0.094, −0.010]), demonstrating statistical significance that corroborates Hypothesis 5b through the interval non-inclusion of zero.

#### 4.4.3. Sequential Mediating Effects

To comprehensively investigate the variable relationships, this research utilized the bootstrapping methodology (PROCESS, Model 6) for sequential mediation analysis. Our approach positioned new employees’ work performance as the independent variable across two distinct models. The initial model examined work engagement as the dependent outcome, incorporating incumbent employees’ perceived status threat and approach-oriented coping as sequential mediators. The secondary model analyzed work withdrawal behavior as the outcome variable, mediated by status threat perceptions and avoidant coping strategies.

The findings presented in Table 5 reveal significant sequential mediation patterns. The mediated pathway involving status threat and approach coping demonstrated an indirect effect of 0.044 (95% CI [0.020, 0.072]), while the sequential mediation effect through status threat and avoidance coping yielded 0.008 (95% CI [0.001, 0.019]). Both confidence intervals excluded zero values, confirming statistical significance. These outcomes substantiate the theoretical framework by illustrating how high-performing new employees elicit status concerns among incumbent employees, consequently shaping work-related behaviors through differential stress response mechanisms.

#### 4.4.4. Moderated Sequential Mediation Effects

This research employed the bootstrapping approach (PROCESS, Model 91) to examine moderated sequential mediation processes (Hayes, 2012). Analyses revealed distinct patterns across the stress mindset conditions. Under low stress mindset conditions, the mediated pathway from new employees’ job performance to work withdrawal through incumbents’ perceived status threat and subsequent avoidance coping demonstrated an effect size of 0.014, supported by a 95% confidence interval (CI = 0.004 to 0.031), excluding zero. Conversely, this sequential mediation mechanism became nonsignificant under high stress mindset conditions (95% CI = −0.011 to 0.006). Crucially, the computed difference between these conditional indirect effects (−0.012) showed statistical significance (95% CI = −0.028 to −0.002). These patterns confirm that the stress mindset operates as a boundary condition moderating the hypothesized mediated relationship, with stronger negative effects observed when employees hold less adaptive stress beliefs. The differential effects across mindset levels provide empirical support for our theoretical propositions regarding psychological responses to workplace transitions.

## 5. Discussion

Drawing on the cognitive appraisal perspective on workplace stress, this study tested a moderated-mediation model linking incumbents’ perceived status threat from high-performing new employees to work engagement and work withdrawal via approach-oriented versus avoidance-oriented coping. Using a two-wave cross-organizational design, the results show that new employees’ exceptional task performance is associated with incumbents’ heightened perceptions of status vulnerability. These perceptions, in turn, display a dual pattern: they are positively related to engagement through approach coping; however, they are also positively related to withdrawal through avoidance coping, a configuration that reflects the co-existence of adaptive and defensive responses to social-evaluative stressors.

Moreover, employees’ stress mindset qualified these relationships. A growth-oriented (enhancing) mindset attenuated the direct link between perceived status threat and avoidance responses and weakened the indirect path from status threat to work withdrawal through avoidance coping, whereas its moderating influence on the relationship between status threat and approach coping was negligible. Interpreted through the appraisal lens, these patterns are consistent with the view that a growth-oriented mindset shapes the secondary appraisal of coping resources, thereby dampening the defensive tendencies without reliably amplifying the approach tendencies under status threat.

### 5.1. Theoretical Contribution

This study advances the theoretical understanding of status threat and its behavioral implications in organizational contexts through three key contributions.

First, it deepens the literature on perceived status threat by identifying new employees’ superior task performance as a novel and empirically supported antecedent of incumbents’ status concerns. Prior studies have primarily examined the outcomes of status threat—such as diminished job satisfaction, reduced organizational commitment, or increased turnover intention (Bai, 2017; Khan et al., 2018; Pettit et al., 2016)—while largely overlooking its situational triggers. By linking new employees’ exceptional performance to incumbents’ sense of status insecurity, this research shifts attention to the origins of perceived status threat and reveals its dual behavioral effects on incumbents: fostering work engagement and fostering withdrawal behaviors. These findings challenge the conventional view of status threat as uniformly detrimental and suggest that its consequences depend on how employees cognitively appraise and manage status-related stressors.

Second, this study enriches the research on new employee assimilation and team adaptation by shifting the analytical lens from new employees’ adjustment processes to incumbents’ adaptive responses. Earlier work on organizational socialization has predominantly focused on how new employees adjust to new environments, build relationships, and develop competencies (Cooper-Thomas et al., 2014; Ellis et al., 2017; Jokisaari & Vuori, 2014; Korte & Lin, 2013). In contrast, this study illuminates how incumbent employees experience and react to status challenges when high-performing new employees enter the workplace. Hence, it integrates the concept of status threat into the socialization literature, providing a more balanced and dynamic account of how both new and incumbent employees contribute to team-level adaptation and relational equilibrium.

Third, the study extends the stress appraisal framework (Lazarus & Folkman, 1984, 1987; Folkman et al., 1986; Bliese et al., 2017) by demonstrating how incumbents evaluate and cope with status-related stressors in the context of social-evaluative challenges. Previous applications of this framework have largely focused on general work stress or performance pressure, without explicitly considering status-based stress as a distinct psychological experience. The present study shows that incumbents’ stress mindset—their generalized belief about whether stress is enhancing or debilitating (Crum et al., 2013)—conditions their cognitive appraisal and behavioral reactions to perceived status threats. This insight expands the boundary of stress theory by linking status dynamics with cognitive-motivational mechanisms, offering a more integrated explanation of how employees interpret and respond to social comparison pressures within organizations.

### 5.2. Practical Implications

This research offers practical guidance for organizational leadership. Initially, it provides leaders with strategies to harness perceived status dynamics effectively. Incumbent employees’ accumulated expertise constitutes a vital competitive asset for enterprises (Schmidt et al., 1986; Paullin, 2014). Institutions should recognize these employees’ contributions while supporting their adaptation to status challenges. Organizations could implement skill development programs to enhance stress perception management, coupled with establishing performance-based rewards or acknowledgment systems. Such initiatives encourage incumbent employees to embrace developmental opportunities during status transitions, rewarding exceptional adaptability (Yin et al., 2024).

Furthermore, the findings enable managers to address potential adverse reactions to status concerns. Supervisory teams should maintain open dialogues with incumbent employees to monitor psychological adjustments, offering appropriate support mechanisms when needed. Companies might implement routine wellness check-ins to reframe status challenges as developmental opportunities rather than stressors (Baer et al., 2010). Cross-generational knowledge transfer initiatives could be developed through collaborative projects, aligning emerging talents’ achievements with incumbent employees’ incentive structures to strengthen collective effectiveness (Liu et al., 2023).

Finally, this investigation supports customized personnel management approaches. Employees demonstrate distinct cognitive patterns affecting their interpretation of status dynamics (Jamieson et al., 2018). Enterprises could integrate stress perception assessments into talent management systems for optimal role alignment. Recruitment processes might incorporate stress response evaluations to match candidates with suitable positions. Given that stress perception represents a dynamic characteristic (Crum et al., 2013, 2017), organizations might implement periodic educational workshops to cultivate adaptive mindsets, ultimately enhancing workforce resilience and productivity.

### 5.3. Limitations and Future Directions

This research shares limitations common to empirical investigations. Primarily, the dataset was collected exclusively through self-assessment surveys from existing staff members, introducing risks of methodological biases inherent in single-source reporting. Furthermore, the observational nature of the investigation restricts definitive conclusions about causation. Subsequent studies could employ experimental designs or multi-wave data collection to explore the causal pathways more effectively. Respondents were drawn from multiple sectors, ensuring cross-industry representation. Nevertheless, we acknowledge that hierarchical norms and performance evaluation systems may vary across industries, which could influence the manifestation of status threat perceptions. Future research should examine these contextual differences to enhance the external validity of the findings.

Regarding theoretical foundations, the cognitive appraisal theory of stress posits that the interpretation of stressors is shaped by both personal characteristics and external conditions. While this investigation focused on individual psychological factors, it did not account for organizational environment factors that might influence reactions to status challenges. Subsequent investigations should incorporate contextual elements such as managerial support systems, collaborative team dynamics, and personnel development strategies when examining responses to perceived status threats.

The current analysis examined performance-driven status competition through a fixed temporal lens. Complementary studies could apply computational modeling techniques to map the evolving relationships between new and incumbent employees. Such dynamic simulations could reveal cyclical patterns in workplace adaptation strategies and emotional responses across different employment phases, potentially informing more responsive personnel management approaches and optimizing organizational interventions.

## Figures and Tables

**Table 1:** Frequency analysis of demographic variables.

Variable	Category	Frequency	Percentage	Variable	Category	Frequency	Percentage
Gender	Male	127	47.74%	Tenure	3–6 years	169	63.53%
	Female	139	52.26%		7–9 years	45	16.92%
Age	18–25	45	16.92%		over 10 years	52	19.55%
	26–35	138	51.88%	Position level	General staff	113	42.48%
	36–45	62	23.31%		Frontline manager	97	36.47%
	46–55	17	6.39%		Department manager	46	17.29%
	>55	4	1.50%		Senior manager	10	3.76%
Education level	Associate’s degree or below	55	20.68%	Organization Type	Government agency/public institution	42	15.79%
	Bachelor’s degree	161	60.53%		State-owned Enterprise	49	18.42%
	Master’s degree	47	17.67%		Private Enterprise	150	56.39%
	Doctoral degree	3	1.13%		Foreign-funded or joint venture enterprise	25	9.40%

Note: N = 266.

**Table 2:** Results of confirmatory factor analysis.

Model	χ^2^	df	CFI	TLI	RMSEA	SRMR
Seven-factor model	486.93	301	0.95	0.95	0.05	0.06
Six-factor model 1 a	788.07	307	0.88	0.86	0.08	0.10
Six-factor model 2 b	879.20	309	0.86	0.84	0.08	0.12
Five-factor model c	1180.69	314	0.79	0.76	0.10	0.14
Four-factor model d	1550.36	318	0.70	0.66	0.12	0.14

Note: a. Combining new employees’ job performance and incumbent employees’ perceived status threat. b. Combining approach coping and avoidance coping. c. Combining new employees’ job performance and incumbent employees’ perceived status threat and combining approach coping and avoidance coping. d. Combining new employees’ job performance and incumbent employees’ perceived status threat, combining approach coping and work engagement, and combining avoidance coping and work withdrawal.

**Table 3:** Means, standard deviations, and correlations.

Variable	M	SD	1	2	3	4	5	6	7	8	9	10	11	12	13
1. Gender	0.52	0.50	-												
2. Age	1.24	0.86	−0.06	-											
3. Education level	0.99	0.66	0.06	−0.18 **	-										
4. Tenure	0.56	0.80	−0.07	0.62 **	−0.09	-									
5. Organization type	1.59	0.87	0.04	−0.01	−0.23 **	−0.13 *	-								
6. Position level	0.82	0.85	−0.10	0.34 **	0.16 **	0.27 **	0.09	-							
7. New Employees’ Job Performance	3.16	0.94	0.04	0.02	0.14 *	−0.05	−0.08	0.10	(0.79)						
8. Incumbent Employees’ Perceived Status Threat	2.17	0.85	−0.06	−0.07	−0.11	−0.08	0.10	−0.14 *	0.24 **	(0.75)					
9. Approach Coping	3.67	0.91	−0.06	−0.09	0.12 *	−0.10	0.11	−0.01	0.19 **	0.48 **	(0.78)				
10. Avoidance Coping	2.41	0.75	0.08	−0.06	0.11	−0.09	−0.01	−0.11	0.05	0.15 *	−0.02	(0.61)			
11. Work Engagement	3.61	0.79	−0.01	0.16 *	0.03	0.16 **	0.07	0.20 **	0.21 **	0.26 **	0.43 **	−0.13 *	(0.74)		
12. Work Withdrawal	1.87	0.56	−0.05	−0.16 **	0.01	−0.08	0.09	−0.23 **	−0.02	0.20 **	−0.02	0.35 **	−0.37 **	(0.62)	
13. Stress Mindset	3.87	0.67	−0.01	0.03	0.05	0.05	−0.03	0.16 *	0.19 **	−0.08	0.18 **	−0.45 **	0.46 **	−0.56 **	(0.71)

Note: ** *p* < 0.010, and * *p* < 0.050. The values in the diagonal brackets represent the arithmetic square roots of the average variance extracted (AVE) for each construct.

**Table 4:** Hierarchical regression analysis results.

	Perceived Status Threat		Work Engagement			Work Withdrawal			Approach Coping			Avoidance Coping		
	M1	M2	M3	M4	M5	M6	M7	M8	M9	M10	M11	M12	M13	M14
Gender	−0.12	−0.14	0.01	0.04	0.07	−0.09	−0.08	−0.11	−0.16	−0.10	−0.09	0.09	0.10	0.10
Age	−0.02	−0.05	0.05	0.06	0.06	−0.08	−0.07	−0.09	−0.03	−0.01	−0.01	0.05	0.04	0.04
Education level	−0.09	−0.13	0.06	0.08	−0.01	0.06	0.07	0.03	0.22 *	0.26 ***	0.26 ***	0.16 *	0.17 *	0.17 **
Tenure	−0.03	0.01	0.10	0.11	0.12	0.06	0.06	0.08	−0.05	−0.04	−0.04	−0.06	−0.05	−0.05
Organization type	0.09	0.12	0.08	0.05	0.02	0.09 *	0.08	0.08 *	0.15 *	0.11	0.10	0.02	−0.01	−0.02
Position level	−0.13	−0.16 *	0.13 *	0.17 **	0.16 *	−0.16 ***	−0.15 ***	−0.12 **	−0.04	−0.01	−0.01	−0.12	−0.04	−0.04
New Employees’ Job Performance		0.26 ***												
Perceived Status Threat				0.28 ***	0.10		0.11 **	0.08 *	−0.16	0.54 ***	0.54 ***		0.11 *	0.13 **
Approach Coping					0.34 ***									
Avoidance Coping								0.24 ***						
Stress Mindset										0.30 ***	0.30 ***		−0.48 ***	−0.46 ***
Stress Mindset × Perceived Status Threat											−0.07			−0.20 **
R^2^	0.04	0.12	0.06	0.15	0.26	0.09	0.11	0.21	0.05	0.32	0.32	0.04	0.24	0.27
ΔR^2^	0.04	0.08	0.06	0.09	0.11	0.09	0.03	0.10	0.05	0.28	0.01	0.04	0.20	0.03

Note: *** *p* < 0.001, ** *p* < 0.010, and * *p* < 0.050.

**Table 5:** Sequential mediating effects.

Dependent Variable		Effect	Boot SE	Boot LL 95% CI	Boot UL 95% CI
Work Engagement	Direct effect	0.10	0.05	0.002	0.190
	Total indirect effect	0.08	0.03	0.025	0.137
	JP–ST–WE	0.02	0.02	−0.011	0.049
	JP–AP–WE	0.02	0.02	−0.024	0.057
	JP–ST–AP–WE	0.04	0.01	0.021	0.075
Work Withdrawal	Direct effect	−0.02	0.04	−0.091	0.047
	Total indirect effect	0.03	0.02	−0.002	0.067
	JP–ST–WW	0.02	0.01	0.001	0.048
	JP–AV–WW	0.01	0.01	−0.024	0.028
	JP–ST–AV–WW	0.01	0.01	0.001	0.018

Note: JP represents new employees’ job performance, ST represents incumbent employees’ perceived status threat, AP represents approach coping, AV represents avoidance coping, WE represents work engagement, and WW represents work withdrawal.

## Data Availability

The raw data supporting the conclusions of this article will be made available by the authors on request.
